# Caffeine affects the biological responses of human hematopoietic cells of myeloid lineage *via* downregulation of the mTOR pathway and xanthine oxidase activity

**DOI:** 10.18632/oncotarget.5212

**Published:** 2015-09-11

**Authors:** Bernhard F. Gibbs, Isabel Gonçalves Silva, Alexandr Prokhorov, Maryam Abooali, Inna M. Yasinska, Maxwell A. Casely-Hayford, Steffen M. Berger, Elizaveta Fasler-Kan, Vadim V. Sumbayev

**Affiliations:** ^1^ School of Pharmacy, University of Kent, Chatham Maritime, ME4 4TB Kent, United Kingdom; ^2^ Department of Pediatric Surgery and Department of Clinical Research, Inselspital, University Hospital, University of Bern, CH-3010 Bern, Switzerland; ^3^ Department of Biomedicine, University of Basel and University Hospital Basel, CH-4031 Basel, Switzerland

**Keywords:** myeloid cells, caffeine, Inflammation, allergy

## Abstract

Correction of human myeloid cell function is crucial for the prevention of inflammatory and allergic reactions as well as leukaemia progression. Caffeine, a naturally occurring food component, is known to display anti-inflammatory effects which have previously been ascribed largely to its inhibitory actions on phosphodiesterase. However, more recent studies suggest an additional role in affecting the activity of the mammalian target of rapamycin (mTOR), a master regulator of myeloid cell translational pathways, although detailed molecular events underlying its mode of action have not been elucidated. Here, we report the cellular uptake of caffeine, without metabolisation, by healthy and malignant hematopoietic myeloid cells including monocytes, basophils and primary acute myeloid leukaemia mononuclear blasts. Unmodified caffeine downregulated mTOR signalling, which affected glycolysis and the release of pro-inflammatory/pro-angiogenic cytokines as well as other inflammatory mediators. In monocytes, the effects of caffeine were potentiated by its ability to inhibit xanthine oxidase, an enzyme which plays a central role in human purine catabolism by generating uric acid. In basophils, caffeine also increased intracellular cyclic adenosine monophosphate (cAMP) levels which further enhanced its inhibitory action on mTOR. These results demonstrate an important mode of pharmacological action of caffeine with potentially wide-ranging therapeutic impact for treating non-infectious disorders of the human immune system, where it could be applied directly to inflammatory cells.

## INTRODUCTION

Caffeine (1,3,7-trimethylxanthine) is a purine alka-loid present in the leaves, seeds or nuts of a number of plants and is consumed by many people worldwide on a daily basis due to its presence in tea or coffee. In humans, caffeine is rapidly demethylated by cytochrome P450 isoform 1A2 and then converted mostly into methylated derivatives of uric acid by the enzyme xanthine oxidase (XOD) [1]. For a long time caffeine was recognised as an isosteric inhibitor of cyclic adenosine monophosphate phosphodiesterase (cAMP-PDE) which upregulates intra-cellular cAMP levels [2]. Caffeine was also found to reduce the role of glycolysis in cell energy metabolism *via* upregulation of lipid degradation (lipolysis) [3, 4].

Recent evidence demonstrated that human hematopoietic cells do not express the cytochrome P450 1A2 isoform and thus should not be able to metabolise caffeine, resulting in the effects of unmodified caffeine [5]. In this case, caffeine could competitively inhibit XOD [6] rather than act as its substrate (most of the other methylxanthines can be converted by XOD). Moreover, several stable purines including caffeine were recently found to inhibit the activity of the mammalian target of rapamycin (mTOR) in somatic cells [7, 8]. It was also demonstrated that at high concentrations (5 mM), caffeine is capable of inhibiting the mTOR pathway in HOS osteosarcoma cells [7]. In addition, 10 mM caffeine was able to inhibit the PI3K/Akt/mTOR/p70S6K pathway in various cell lines including SH-SY5Y neuroblastoma cells and HeLa cells [9]. 10 mM caffeine was even capable of inhibiting the phosphorylation (Ser473) of Akt in SH-SY5Y cells [9]. In myeloid cells mTOR, a highly conserved serine/threonine kinase, acts as a central regulator of cell growth and metabolism and plays crucial pathophysiological roles in host immune defence, allergic reactions and leukaemia [10]. Importantly, the mTOR pathway plays a pivotal role in non-hypoxic activation of the hypoxia-inducible factor 1 (HIF-1) transcription complex in human myeloid cells. HIF-1 controls the expression of over 40 target genes responsible for glycolysis, angiogenesis and cell adhe-sion – physiological processes which form a critical part of myeloid cell function in the human immune system. This transcription complex, containing an inducible α and a constitutive β subunit, is a major component of the myeloid cell stress adaptation machinery [11, 12]. Therefore, inhibiting the mTOR/HIF-1 metabolic/signalling axis could be an excellent therapeutic strategy for treating human disorders associated with myeloid cell function – leukaemia, autoimmune disease, and allergy. However, existing mTOR inhibitors are toxic and can cause major side effects and adverse drug reactions. Thus, if the inhibitory activity of caffeine on mTOR has indeed been overlooked for decades, this agent may be an excellent non-toxic drug candidate for the correction of pathophysiological responses of human hematopoietic cells of myeloid lineage.

Here we report for the first time that caffeine inhibits the activation of mTOR in THP-1 human myeloid leukaemia cells, primary human acute myeloid leukaemia (AML) cells and primary human basophils. In THP-1 and primary AML cells caffeine was also found to inhibit XOD. In all cases, the caffeine-mediated attenuation of the mTOR pathway led to the downregulation of ligand-induced glycolysis and cytokine/growth factor/mediator production. Caffeine is known to upregulate lipolysis through activation of hormone sensitive lipase (HSL). This upregulates the Krebs’ cycle leading to decreased intracellular levels of 2-oxoglutarate (2-OG), thus preventing degradation of HIF-1α protein (the inducible HIF-1 subunit) by a classical mechanism controlled by HIF-1α prolyl hydroxylases (PHDs). This effect was observed in all of the myeloid cell types studied except for basophils, where HIF-1α accumulation was less PHD-dependent and caffeine completely blocked IgE-induced HIF-1α accumulation. High performance liquid chromatography (HPLC) experiments demonstrated that caffeine entered all of the above cell types and was not metabolised. Taken together, our results reveal novel mechanisms for the downregulatory effects of caffeine on the biological responses of human myeloid cells.

## RESULTS

### Caffeine inhibits ligand-induced activation of the mTOR pathway and its downstream effects in THP-1 human AML cells

**Figure 1:**
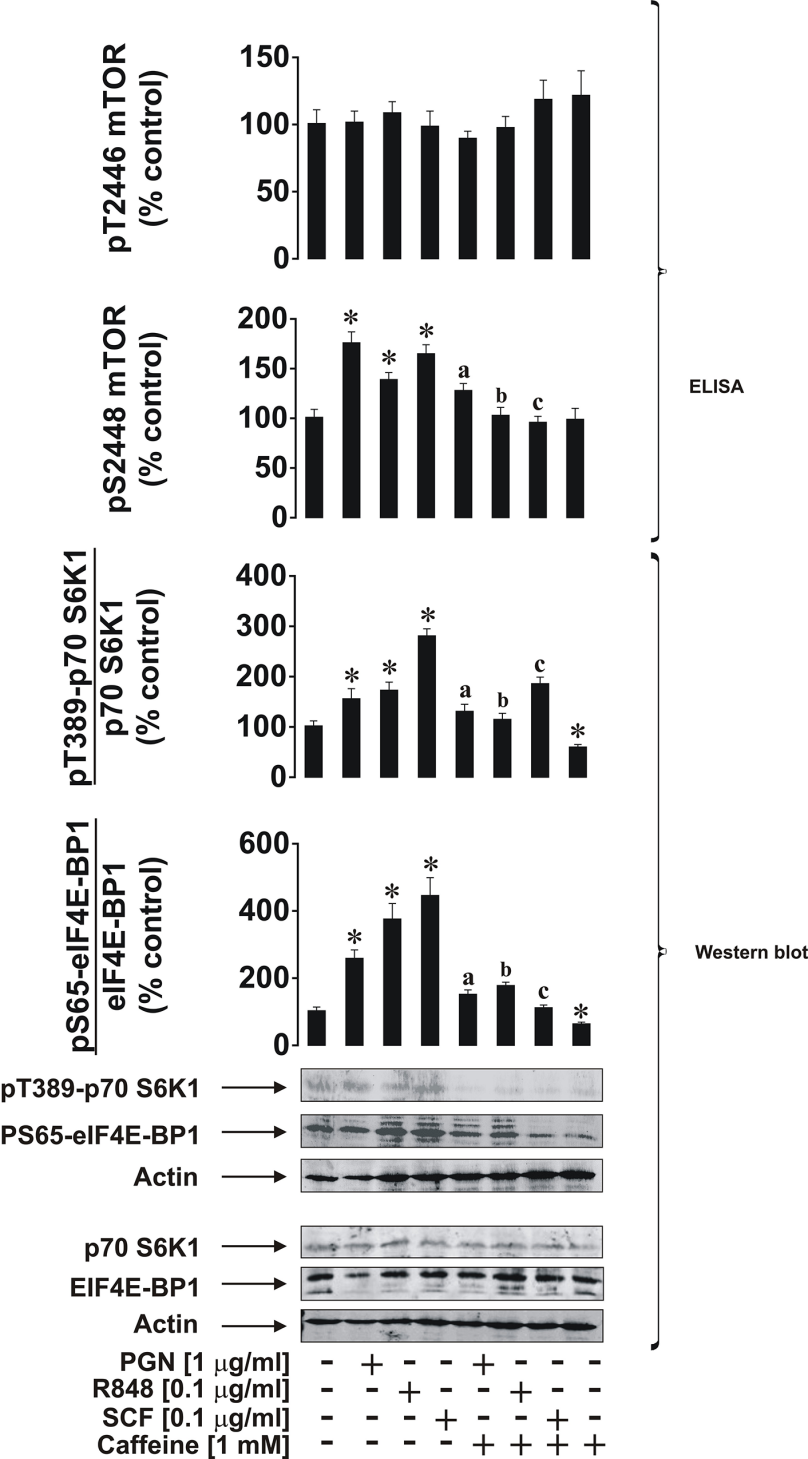
Caffeine inhibits the ligand-induced mTOR signalling pathway in THP-1 human acute myeloid leukaemia cells Cells were exposed for 4 h to the indicated concentrations of PGN, R848 or SCF with or without 1 h pre-treatment with 1 mM caffeine. Levels of pS2448 and pT2446 phospho-mTOR as well as phosphorylation of its downstream enzymes were analysed as outlined in the Materials and Methods. Western blot data show one representative experiment of four that gave similar results and were quantitatively analysed. Quantitative data are shown as means ± S.D; * – *p* < 0.01 vs. control (*n* = 4), a – *p* < 0.01 vs. PGN alone, b – *p* < 0.01 vs. R848 alone and c – *p* < 0.01 vs. SCF alone.

We first investigated the effects of caffeine on ligand-induced mTOR activation through phosphorylation of its S2448 residue in THP-1 cells. Cells were exposed for 4 h to ligands (see below) with or without 1 h pre-treatment with 1 mM caffeine (this concentration corresponds to a therapeutic dose of caffeine and is well below the toxic dose [13]). In line with our previous observations [10], we found that pro-inflammatory ligands of Toll-like receptors (TLRs) 2 (plasma membrane-associatedTLR – 1 μg/ml peptidoglycan (PGN) was used as a ligand), 7/8 (endosomal TLRs recognising viral single-stranded RNA – 0.1 μg/ml resiquimod (R848) was employed as a ligand) induce activating S2448 phosphorylation of mTOR. The same effect was observed for stem cell factor (SCF), a major hematopoietic factor which is also known to promote growth of leukaemia cells, where caffeine (1 mM) attenuated its effect in all cases (Figure 1). Intriguingly, caffeine alone did not significantly reduce phospho-S2448 mTOR in non-stimulated cells but significantly reduced background levels of phospho-T389 p70 S6K1 and phospho-S65 eIF4E-BP1 (see Figure 1 for details). This suggests that caffeine is likely to act directly on mTOR as well, which would prevent further phosphorylation but not impact on existing activity. The presence of caffeine close to the catalytic site (in the active site of mTOR) would almost certainly affect its kinase activity. However, it is important to stress that phosphorylation of mTOR at the inhibitory T2446 site was not increased by caffeine. This observation clearly suggests that caffeine is unlikely to enhance the activity of this mTOR downregulatory pathway and is likely to be a potential direct mTOR inhibitor, as some other purine containing compounds (such as cAMP) do [8]. Phosphorylation levels of its substrates (indicators of mTOR kinase activity) – p70 S6 kinase 1 (p70 S6K1 – at position T389) and eukaryotic initiation factor 4E-binding protein 1 (eIF4E-BP1 at position S65) were also significantly upregulated by all the employed ligands as well as phosphorylation of S2448 mTOR. All these processes were attenuated by caffeine (Figure 1).

**Figure 2:**
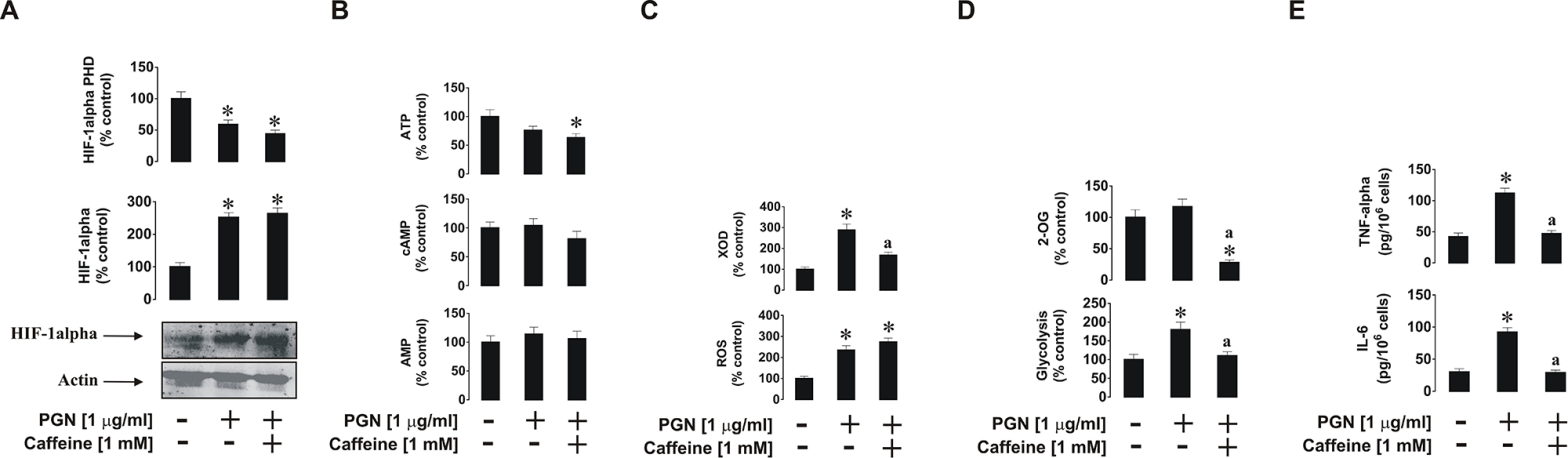
Effects of caffeine on TLR2-mediated biological responses of THP-1 cells Cells were exposed for 4 h to PGN with or without 1 h pre-treatment with 1 mM caffeine before assessing the indicated biological responses: **A.** HIF-1α accumulation, **B.** ATP/cAMP/AMP levels, **C.** XOD activity and intracellular ROS pool, **D.** glycolysis and 2-OG level as well as **E.** TNF-α/IL-6 release. Western blot data show one representative experiment of three that gave similar results. Quantitative data are shown as means ± S.D; * – *p* < 0.01 vs. control (*n* = 3), a – *p* < 0.01 vs. PGN alone.

We next investigated the effects of caffeine on mTOR downstream and associated biochemical pathways induced by PGN, R848 and SCF. We found that caffeine did not downregulate PGN-induced accumulation ofHIF-1α protein. This was in line with a significant decrease in HIF-1α PHD activity in cells treated with PGN in the presence of caffeine (Figure 2A). ATP levels, which are often controlled in a HIF-1-dependent manner, were decreased in cells co-stimulated with PGN and caffeine. Levels of cAMP did not undergo significant changes, probably owing to the fact that receptors involved in these events are not G-protein-coupled [14]. They do not provoke adenylate cyclase activation/cAMP production and thus cAMP-PDE-inhibiting activity of caffeine has no effect in this case. AMP levels were also not increased by PGN stimulation, which conforms with our previous observations (Figure 2B). The presence of caffeine did not increase AMP levels despite a significant decrease in PGN-induced XOD activity (Figure 2C). This is likely to be caused by the inhibition of hypoxanthine-guanine phosphoribosyl transferase (HGPRT), an enzyme which converts hypoxanthine (accumulated when XOD was inhibited) into inosine monophosphate (IMP), which is then converted into AMP [15, 16]. However, we could not rule out the possibility of caffeine-dependent effects on other enzymes involved in the conversion of hypoxanthine into AMP. Intracellular reactive oxygen species (ROS) levels upregulated by PGN were also unaffected by caffeine, possibly because XOD is not the main contributor to the intracellular ROS pool (NADPH oxidase plays this role [17]) in this case (Figure 2C). Glycolysis, which was significantly upregulated by PGN, was reduced to its basic level (Figure 2D). This is not surprising, since translation of glycolytic enzymes is likely to be an mTOR-dependent process in human hematopoietic cells [18]. However, intracellular 2-OG levels were almost completely abolished by caffeine (Figure 2D). This may be due to the caffeine-dependent upregulation of lipolysis and, therefore, the Krebs’ cycle (which would lead to a reduction of intracellular 2-OG levels and a subsequent decrease in 2-OG-dependentHIF-1α PHD activity, where 2-OG is used as a cofactor [19]; which we observed (Figure 2A)). Furthermore, the pro-inflammatory responses of THP-1 cells induced by PGN (release of TNF-α and IL-6) were attenuated by caffeine (Figure 2E).

**Figure 3:**
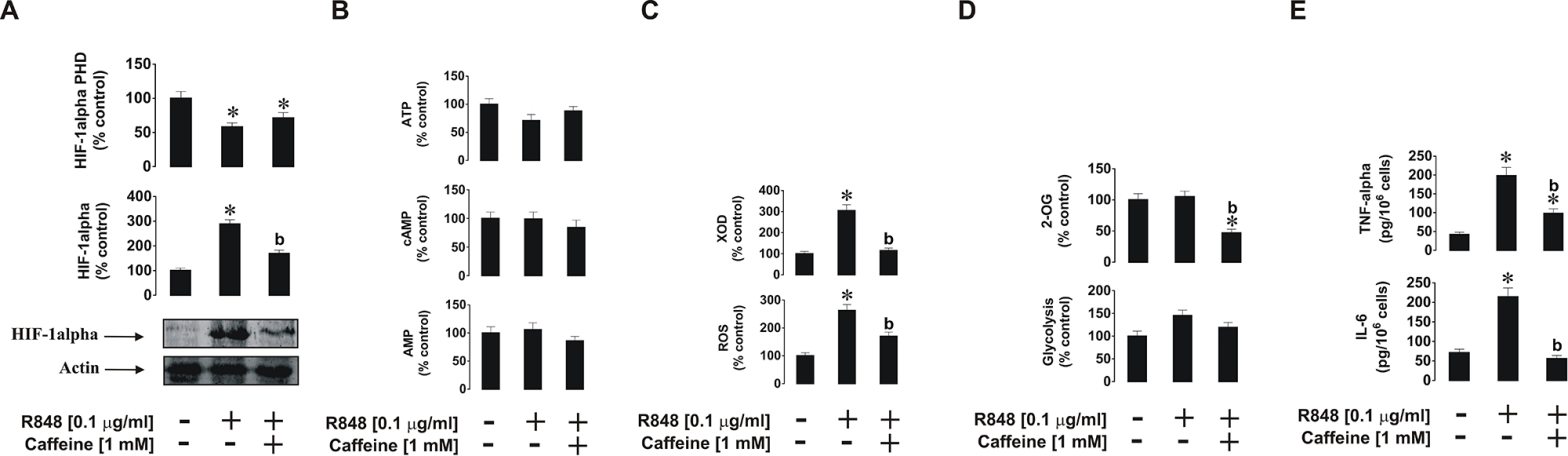
Effects of caffeine on TLR7/8-mediated biological responses of THP-1 cells Cells were exposed for 4 h to R848 with or without 1 h pre-treatment with 1 mM caffeine before analysing **A.** HIF-1α accumulation, **B.** ATP/cAMP/AMP levels, **C.** XOD activity and intracellular ROS pool, **D.** glycolysis and 2-OG level as well as **E.** TNF-α/IL-6 release. Western blot data show one representative experiment of three that gave similar results. Quantitative data are shown as means ± S.D; * – *p* < 0.01 vs. control (*n* = 3), b – *p* < 0.01 vs. R848 alone.

The situation with endosomal TLRs 7 and 8 was slightly different, where THP-1 cells were exposed to 0.1 μg/ml R848 with or without 1 h pre-treatment with 1 mM caffeine. We found that, unlike with PGN, R848-induced HIF-1α accumulation was significantly reduced by caffeine. HIF-1α PHD activity was significantly decreased by R848 (as we have reported before [20]), but in the presence of caffeine, PHD activity did not reduce further and was even slightly elevated compared to exposure to R848 alone (Figure 3A). ATP, AMP and cAMP levels in the cells were not affected by caffeine as in the experiments with PGN (Figure 3B). Caffeine inhibited R848-induced XOD activity but also (unlike following the exposure of THP-1 cells to PGN) it reduced intracellular ROS levels upregulated by R848. In the case of endosomal TLRs, XOD contributes to the intracellular ROS pool [17] together with NADPH oxidase (Figure 3C). This result explains the caffeine-dependent decrease inHIF-1α accumulation in THP-1 cells induced by R848, since ROS may play a primary role in the process. Glycolysis was also significantly upregulated in the presence of R848 and this effect was slightly reduced by caffeine. 2-OG levels were significantly reduced (by *ca*. 50%) by caffeine (Figure 3D). R848-induced production of IL-6 and TNF-α were significantly lower in the presence of caffeine (Figure 3E).

**Figure 4:**
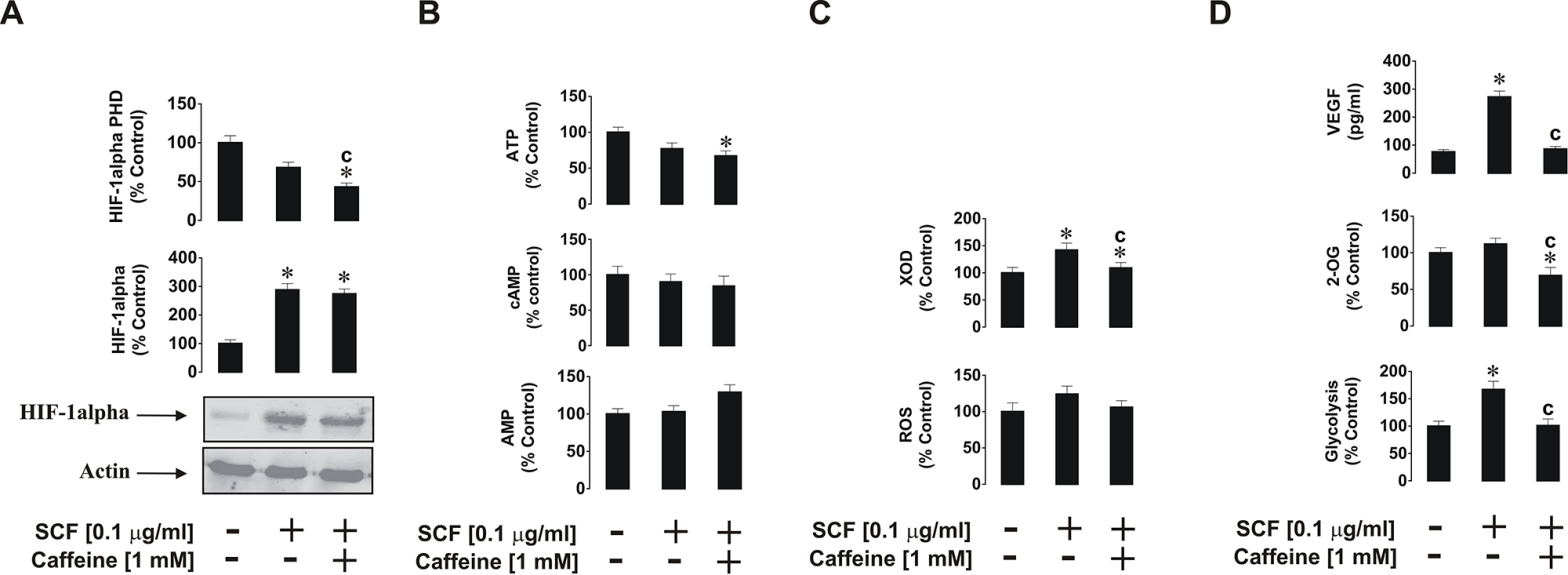
Effects of caffeine on SCF-induced Kit receptor activation in- THP-1 cells Cells were exposed for 4 h to SCF with or without 1 h pre-treatment with 1 mM caffeine before analysing **A.** HIF-1α accumulation, **B.** ATP/cAMP/AMP levels, **C.** XOD activity and intracellular ROS pool, **D.** glycolysis and 2-OG level as well as VEGF release. Western blot data show one representative experiment of three that gave similar results. Quantitative data are shown as means ± S.D; * – *p* < 0.01 vs. control (*n* = 3), c – *p* < 0.01 vs. SCF alone.

SCF-induced biological responses of THP-1 cells were also affected in a way similar to that observed for PGN (see above). THP-1 cells were exposed for 4 h to 0.1 μg/ml SCF with or without 1 h pre-treatment with 1 mM caffeine. We found that SCF-induced HIF-1α accumulation was not decreased in the presence of caffeine which corresponded to a striking decrease in HIF-1α PHD activity in the cells (Figure 4A). ATP, cAMP and AMP levels were not affected at all (Figure 4B). As with TLRs, the Kit receptor, which recognises SCF as a ligand, is not a G-protein coupled receptor (it is a typical receptor tyrosine kinase), and therefore no changes in cAMP levels were observed. SCF-induced XOD activity was reduced by caffeine, however intracellular ROS levels were slightly upregulated by SCF and remained unchanged in the presence of caffeine (Figure 4C). SCF also upregulated glycolysis and caffeine attenuated this process as well as significantly reduced intracellular 2-OG levels (Figure 4D). Finally, SCF-induced vascular endothelial growth factor (VEGF) production was significantly increased upon exposure of the cells to SCF, but this process was attenuated by caffeine (Figure 4D).

**Figure 5:**
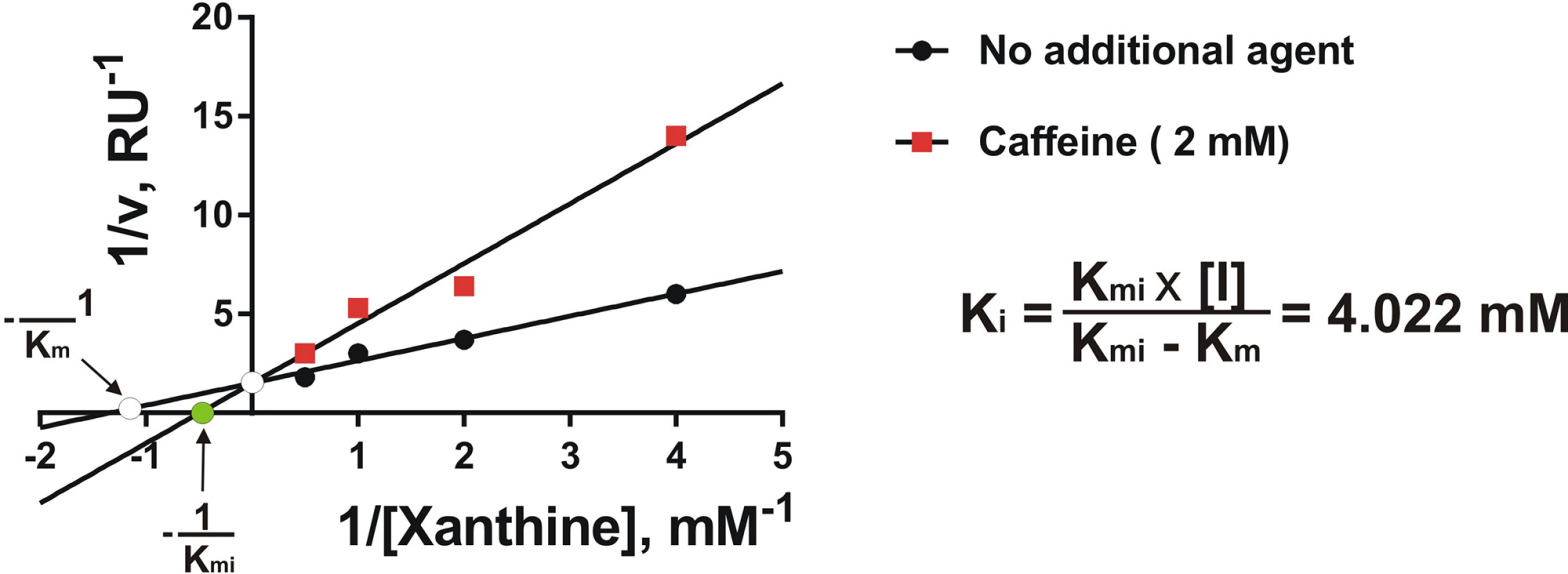
Caffeine is a weak competitive inhibitor of XOD Kinetics of the effects of caffeine on the activity of purified bovine liver XOD were analysed as described in the Materials and methods. Each point was analysed in quadruplicate.

In order to confirm that caffeine is able to directly inhibit XOD and, if so, to characterise this process, we studied the kinetics of caffeine effects on purified bovine liver XOD activity using the Lineweaver-Burk approach [21]. We found that caffeine isosterically (in a competitive manner) inhibited XOD (Figure 5). However, the Ki was only 4.022 mM suggesting that caffeine is a weak inhibitor compared to classic XOD inhibitors (for example, allopurinol and sodium tungstate). This confirmed our previous studies showing the ability of caffeine to inhibit XOD *in vitro* but not *in vivo* due to demethylation of the drug.

### Caffeine affects SCF-induced responses of primary human AML cells in an mTOR-dependent manner

**Figure 6:**
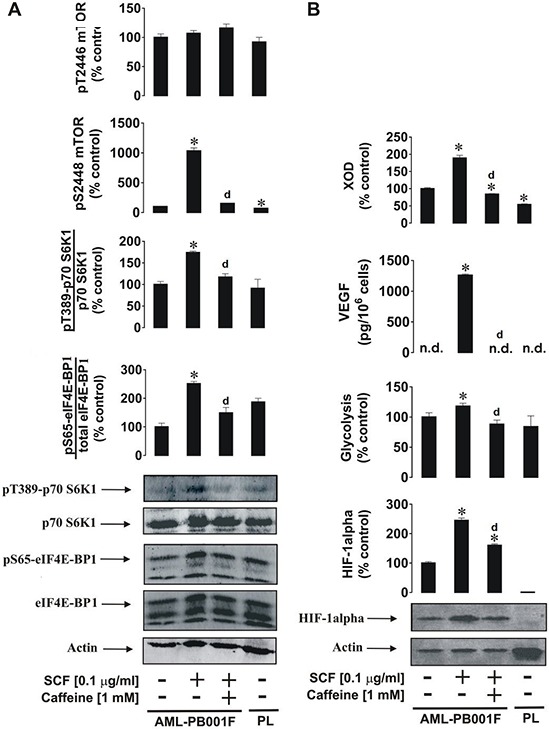
Effects of caffeine on SCF-induced Kit receptor activation in primary human AML cells AML-PB001F cells were exposed for 4 h to SCF with or without 1 h pre-treatment with 1 mM caffeine before analysis of **A.** mTOR phosphorylation and its biological activity and **B.** HIF-1α accumulation, glycolysis, VEGF release and XOD activity. Western blot data (50,000 cells per well were loaded in each case for AML cells and 90,000 for healthy primary leucocytes) show one representative experiment of three-four (using cells derived from different donors) that gave similar results. In both panels A and B we also show the results obtained from non-treated healthy primary human leukocytes (PL) handled as described in Materials and Methods. Quantitative data are shown as means ± S.D; * – *p* < 0.01 vs. control (*n* = 4 for panel A and *n* = 3 for panel B), d – *p* < 0.01 vs. SCF alone.

We sought confirmation of our results using primary human leukocytes. For this purpose we used primary acute myeloid leukaemia cells AML-PB001F purchased from AllCells (Alameda, CA, USA). Cells were exposed to 0.1 μg/ml SCF (these cells express high levels of the Kit receptor) for 4 h with or without 1 h pre-treatment with 1 mM caffeine. We found that SCF induced a significant increase in mTOR phosphorylation at position S2448 and respectively phosphorylation of its substrates – p70 S6K1 and eIF4E-BP1 (Figure 6A). These levels were compared with those in resting primary human leukocytes (PL) obtained from healthy donors (Figure 6A). As in THP-1cells, SCF induced significant upregulation of HIF-1α accumulation, glycolysis, VEGF release and XOD activity. All these effects were attenuated by caffeine (in the case of HIF-1α – downregulated; the level was still significantly higher than the control) (Figure 6B). Interestingly, background levels of HIF-1α, pS2448 mTOR and XOD activity were significantly higher in primary AML cells compared to primary “healthy” leukocytes (PL) suggesting that these factors/pathways are crucial for AML cell function. Importantly, resting primary human leukocytes obtained from the blood of healthy donors did not accumulate detectable amounts of HIF-1α protein.

**Figure 7:**
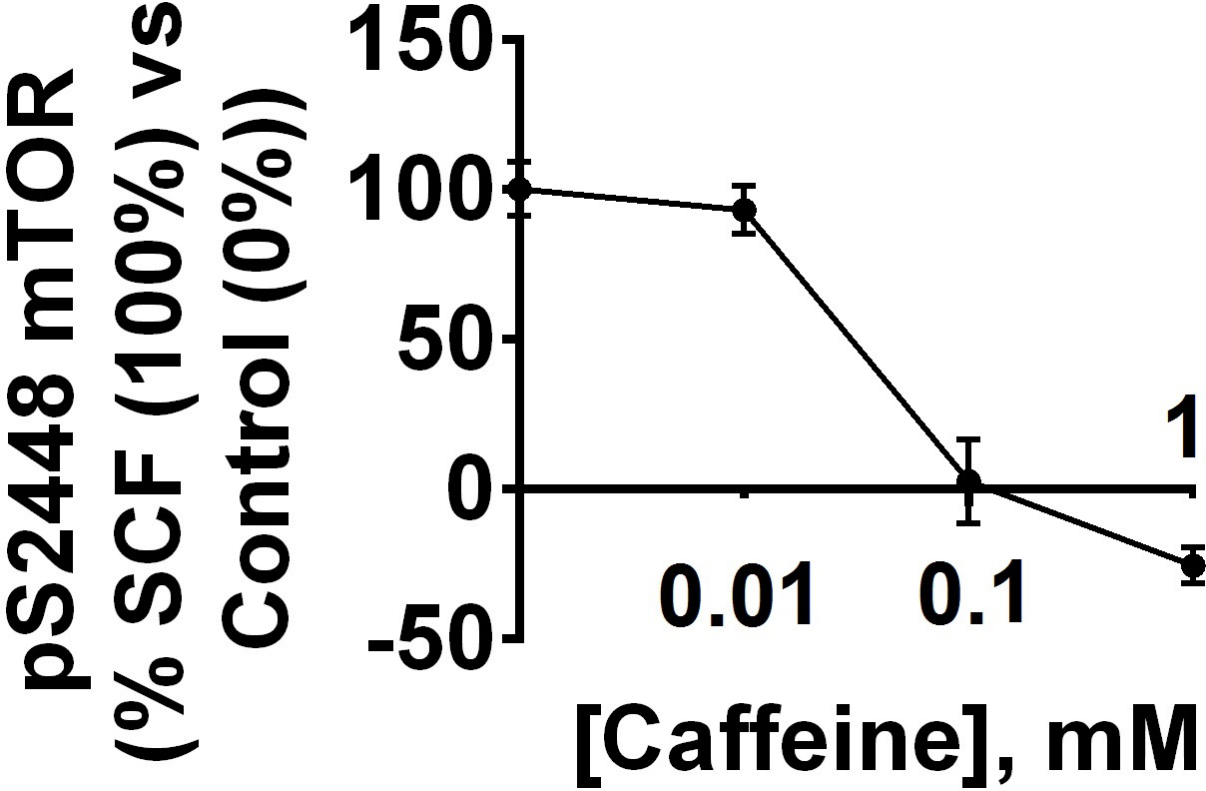
Dose-dependent effects of caffeine on SCF-induced Kit receptor-mediated intracellular pS2448 mTOR levels AML-PB001F cells were exposed for 4 h to 0.1 μg/ml of SCF with or without 1 h pre-treatment with 0.01, 0.1 or 1 mM caffeine. pS2448 mTOR levels were analysed. Quantitative data are shown as means ± S.D of three individual experiments; * – *p* < 0.01 vs. control.

Given the effects of caffeine on AML cell function, we next determined the concentration-dependent actions of caffeine on these cells following exposure to 0.1 μg/ml SCF for 4 h with or without 1 h pre-treatment with 0.01, 0.1 and 1 mM caffeine. Levels of pS2448 mTOR were monitored as a biochemical response. We found that SCF-dependent mTOR phosphorylation was significantly affected by 1 and 0.1 but not 0.01 mM caffeine (Figure 7).

### Caffeine downregulates anti-IgE-induced activation of human basophils by affecting mTOR signalling

**Figure 8:**
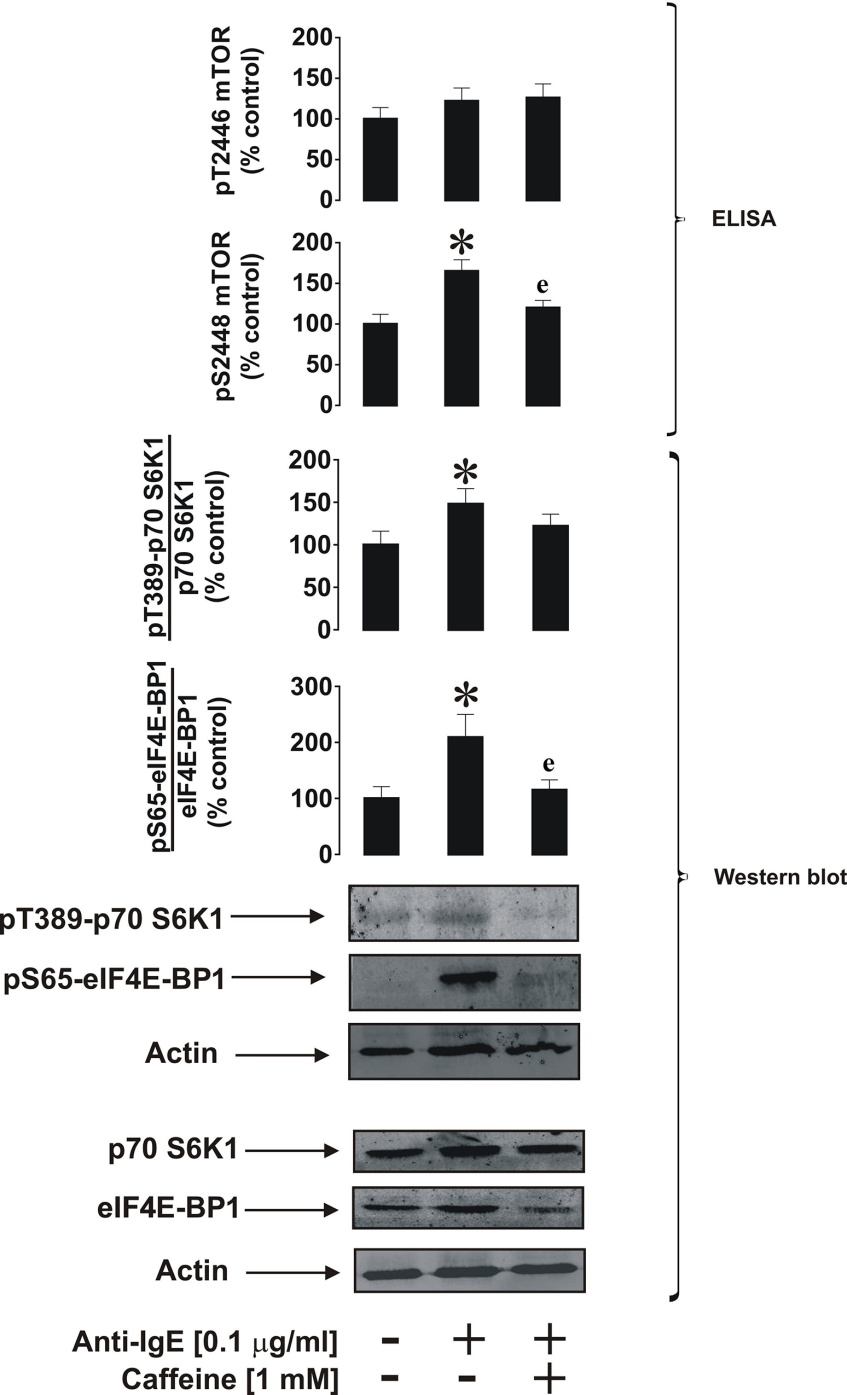
Caffeine inhibits ligand-induced mTOR signalling pathway in primary human basophils Cells were exposed for 2 h to 0.1 μg/ml anti-IgE with or without 30 min pre-treatment with 1 mM caffeine. pS2448 and pT2446 phospho-mTOR levels as well as phosphorylation of its downstream enzymes were analysed as described in Materials and Methods. Western blot data show one representative experiment of four that gave similar results. Quantitative data are shown as means ± S.D; * – *p* < 0.01 vs. control (*n* = 4), e – *p* < 0.01 vs. anti-IgE alone.

We were next interested in investigating the effects of caffeine on primary human basophils. These terminally differentiated granulocytes display biochemically similar but pathophysiologically different responses compared to THP-1 and primary AML cells. Furthermore, basophils play a crucial role in human allergic reactions since they readily respond to IgE-dependent triggering, although compared to leukaemic myeloid cells they are relatively unresponsive to SCF [22] and TLR2/4 ligands [23]. Stimulation of primary human basophils with 1 μg/ml anti-IgE led to the activation of mTOR phosphorylation at S2448, which was attenuated by caffeine (Figure 8). Similar effects were observed regarding the phos-phorylation of mTOR substrates (Figure 8).

**Figure 9:**
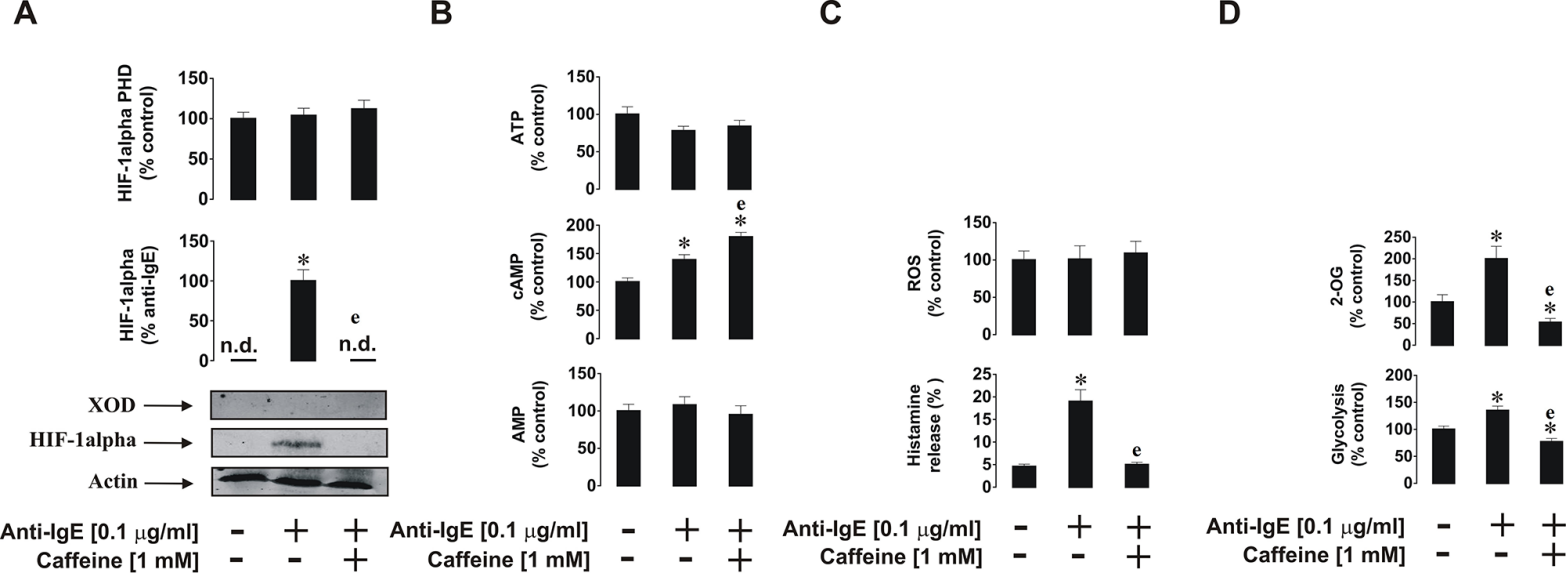
Effects of caffeine on the anti-IgE-induced biological responses of primary human basophils Cells were exposed for 2 h to 0.1 μg/ml anti-IgE with or without 30 min pre-treatment with 1 mM caffeine before analysis of **A.** HIF-1α accumulation and XOD protein levels, **B.** ATP/cAMP/AMP levels, **C.** intracellular ROS pool and histamine release, **D.** glycolysis and 2-OG level. Western blot data show one representative experiment of three that gave similar results. Quantitative data are shown as means ± S.D ; * – *p* < 0.01 vs. control (*n* = 3), e – *p* < 0.01 vs. anti-IgE alone.

As shown previously [24], HIF-1α accumulation was upregulated by anti-IgE. This process was completely blocked by caffeine and the process appeared to be independent of PHD activity, since no significant changes were observed in enzyme activity (Figure 9A). Importantly, we could not detect any XOD activity in basophils, which is supported by our previous observations [21]. Western blot analysis (Figure 9A) demonstrated the absence of XOD protein in basophils. ATP and AMP levels were not affected by any of the treatments; however, cAMP levels were significantly upregulated by anti-IgE and were further increased in the presence of 1 mM caffeine (Figure 9B). This suggests that IgE-induced responses in basophils are indirectly linked to G-protein-coupled receptor signalling, possibly due to the actions of histamine acting through H2 receptors, which may serve as a negative feedback loop limiting further basophil degranulation and histamine release. Intracellular ROS levels were not affected which confirms our previous findings suggesting that IgE-induced basophil responses are orchestrated in a redox-independent manner (Figure 9C). IgE-dependent histamine release was significantly affected by caffeine which is in line with the observed caffeine-dependent downregulation of IgE-induced glycolysis and 2-OG levels (Figure 9D). However, the effect associated with 2-OG levels did not impact HIF-1α accumulation or PHD activity, further confirming our hypothesis that this process is most likely to be PHD-independent in basophils.

We next further verified the differential involvement of XOD in the biological responses of myeloid cells by assessing whether basophil function could be affected by XOD inhibitors/substrate. We studied the effects of the XOD inhibitor allopurinol, the XOD sub-strate hypoxanthine (hypoxanthine – chemically is an allopurinol isomer) and the highly specific XOD inhibitor sodium tungstate. Neither of these compounds, however, was able to significantly affect anti-IgE-stimulated basophil histamine release and HIF-1α accumulation (Supplementary Figure S1). We also noticed that, while 0.01 mM caffeine did not significantly inhibit IgE-induced histamine release, HIF-1α accumulation was still markedly reduced at this low caffeine concentra-tion (Supplementary Figure S1). This provides further confirmation of our previous observations [24] suggesting that histamine release and HIF-1 activation are controlled by biochemically independent pathways.

### Caffeine is taken up but not metabolised by human myeloid cells

**Figure 10:**
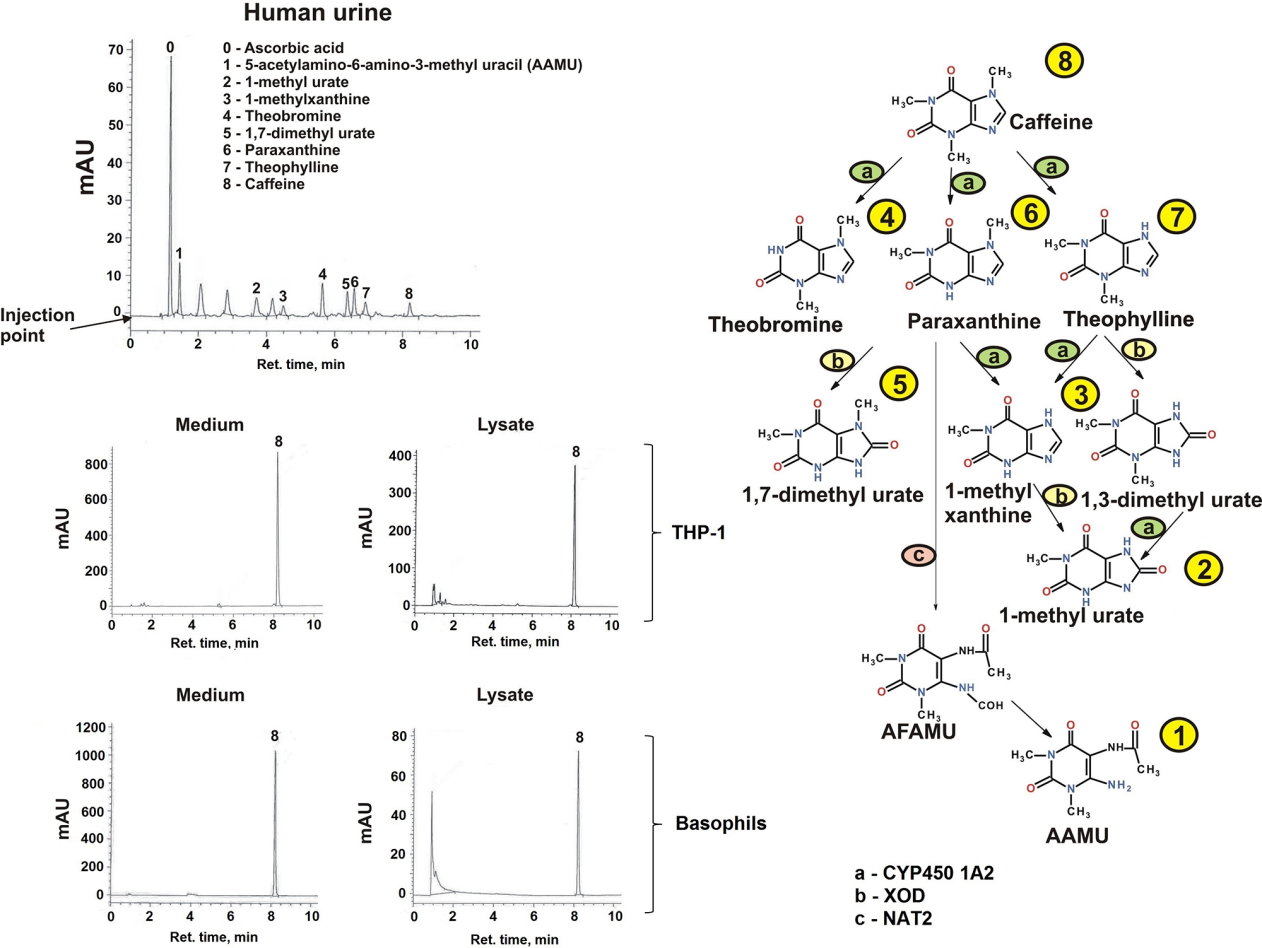
Determination of caffeine and its metabolites in human urine and myeloid cells Caffeine and its metabolites in human urine (coffee drinkers) and medium/lysates of THP-1 cells (exposed for 4 h to 1 mM caffeine) and human basophils (exposed for 2 h to 1 mM caffeine) were detected by HPLC. The caffeine metabolic pathway in humans is also presented indicating how each metabolite is obtained biochemically. CYP4501A – cytochrome P450 A2; XOD – xanthine oxidase; NAT2 – N-acetyltransferase 2; AAMU – 5 Acetylamino-6-amino-3-methyluracil; AFMU – 5 Acetylamino-6-formylamino-3-methyluracil.

In order to ascertain whether the observed effects described above were due to caffeine or a metabolite we investigated whether the different myeloid cell types employed in the study could metabolised caffeine following cellular uptake. We found that neitherTHP-1 cells nor primary human leukocytes nor basophils contained the cytochrome P450 1A2 isoform which is primarily responsible for caffeine demethylation (data not shown since they are at zero levels). We then assessed whether caffeine (a) enters the studied cells and (b) is metabolised through other pathways. For this purpose we used high-performance liquid chromatography (HPLC) in order to detect caffeine and its metabolites. As a positive control we used urine of healthy volunteers following consumption of caffeine by them. In the urine we detected six caffeine metabolites as well as unmodified caffeine, suggesting that all the metabolites are detectable by our method and that caffeine is mostly metabolised *in vivo* in humans (Figure 10). In both THP-1 cells and primary human basophils we detected caffeine but none of its metabolites, suggesting that caffeine is endocytosed into these myeloid cells but is not further metabolised. This finding was confirmed by the fact that none of the caffeine metabolites was detected in the culture medium; only unmodified caffeine was detected (Figure 10). These data suggest that the effects of caffeine observed in the study were solely caused by unmodified caffeine.

Taken together, our results demonstrate for the first time that caffeine affects the biological responses of human myeloid cells, including leukaemia cell lines, primary AML cells and primary human basophils by downregulating the mTOR pathway and differentially inhibiting XOD.

## DISCUSSION

Caffeine, a plant-derived purine alkaloid, was chemically identified over 100 years ago and its effects were observed even before Biochemistry developed into an independent field of science [1]. However, due to its metabolism and labile properties, the effects of caffeine in human hematopoietic cells (where it remains unmetabolised) were completely overlooked. Given the fact that some purine compounds were found to directly inhibit mTOR, we were interested to see whether caffeine displays such an activity too. Alternatively, unmodified caffeine was found to inhibit XOD, which we recently showed to play a major role in myeloid cell responses. We were therefore interested in discovering whether caffeine plays a major role in the biological responses of human hematopoietic cells of myeloid lineage based on its potential actions on XOD and mTOR. These leukocytes determine human innate immune responses and are affected during severe human disorders such as AML and allergy. Thus, elucidating a potentially overlooked mechanism of caffeine may result in additional therapeutic uses for this methylxanthine which was rapidly taken up by these myeloid cells but not metabolised.

We analysed the effects of caffeine on the pro-inflammatory and pro-leukaemic responses of human AML cells using THP-1 cell line and primary AML cells as well as on the pro-allergic reactions of primary human basophils. Caffeine was found to downregulate activating phosphorylation (S2448 position) of the mTOR protein thus affecting its kinase activity. This occurred in both THP-1 cells (upon exposure to PGN, R848 and SCF) as well as in primary AML cells stimulated with SCF (TLR expression levels are quite low in these cells). Importantly, caffeine did not increase phosphorylation of the T2446 residue of mTOR. This shows that caffeine does not activate specific mTOR downregulatory cascades, for example the AMPK pathway [10]. Intracellular AMP (specific AMP kinase activator) levels were also not affected by caffeine. However, caffeine inhibited XOD activity in both THP-1 and primary AML cells. Our previous studies showed that inhibition of XOD by allopurinol or sodium tungstate led to a significant increase in intracellular AMP levels and therefore induced AMPK-dependent downregulatory phosphorylation of mTORat position T2446 [21]. Here, we also observed inhibition of XOD but without increases in intracellular AMP levels. Recently, it was reported that caffeine is capable of inhibiting Akt phosphorylation thus preventing mTOR activation (presumably through phosphorylation of its S2448 residue). However, other studies demonstrated that, in tissues expressing high levels of XOD (for example skeletal muscles), high concentrations of caffeine (10 mM) activate AMPK [7]. These tissues, however, can metabolise caffeine. XOD expression in leukocytes (even in AML cells) is relatively low compared to other tissues and in basophils where the presence of XOD was even undetectable [21].

Although caffeine is a weak inhibitor of XOD compared to allopurinol and especially sodium tungstate, it might affect the re-use of purines in myeloid cells (this system is quite robust in leukocytes and is not affected by allopurinol or tungastates, which are specific XOD inhibitors only – especially tungstates, where tungsten replaces molybdenum in the catalytic site of the enzyme causing its irreversible inhibition). Caffeine is known to inhibit HGPRT, however, in myeloid cells its inhibitory effects are more striking since it is not metabolised in these cells [25]. This means that, upon inhibition of XOD by caffeine, levels of hypoxanthine are increased. However, hypoxanthine can't be converted into IMP and further down to AMP [21] (for more details, see Supplementary Figure S2). This lack of increase in AMP levels prevents the activation of AMP kinase and subsequent phosphorylation of mTOR at T2446.

**Figure 11:**
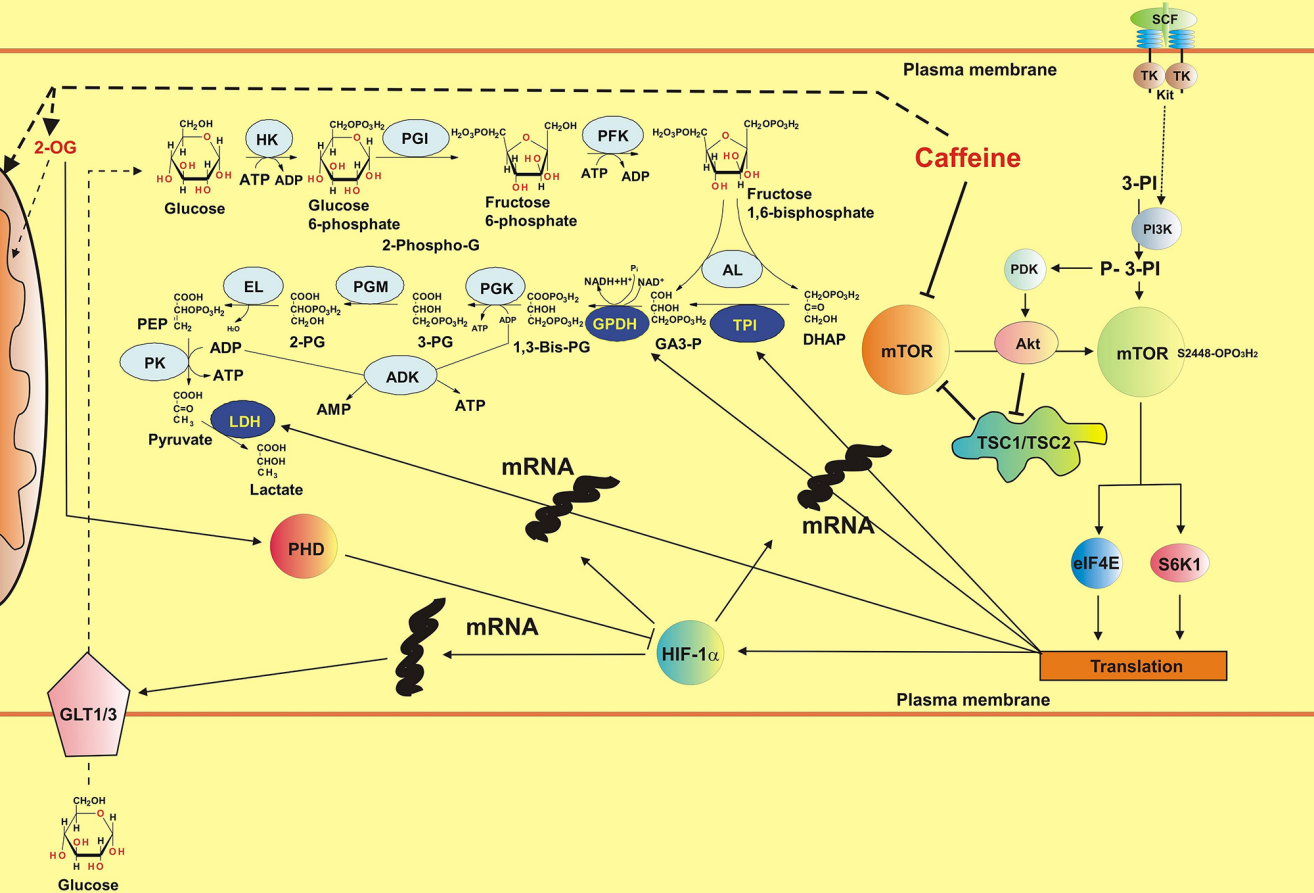
Possible biochemical mechanisms of the effects of caffeine on the mTOR pathway, HIF-1 activity and energy metabolism in human myeloid cells (SCF-induced responses are used as the example) Additional abbreviations used: HK – hexokinase, PGI – phosphoglucose isomerase, PFK – phosphofructokinase, AL – Aldolase, TPI – Triose-phosphate isomerase, GPDH – glyceraldehyde-3-phosphate dehydrogenase, PGK – phosphoglycerate kinase, PGM – phosphoglycerate mutase, EL – enolase, PK – pyruvate kinase, LDH – lactate dehydrogenase, DHAP – dihydroxyacetone phosphate, GA3-P – glyceraldehyde-3-phosphate, 3-PG – 3-phosphoglycerate, 2-PG – 2-phosphoglycerate, PEP – phosphoenolpyruvate, GLT1/3 – glucose transporters 1/3, TSC1/TSC2 – Tuberous sclerosis proteins 1 and 2, PDK – P-3-PI-dependent kinase, ADK – adenylate kinase, TK – tyrosine kinase.

Inhibition of mTOR and XOD by caffeine did not lead to the attenuation of HIF-1α accumulation inTHP-1 and primary AML cells. This was due to a significant decrease in HIF-1α PHD activity (resulting from the decrease in intracellular 2-OG levels required for this reaction in form of a co-factor). Caffeine activates lipolysis by upregulating the activity of hormone-sensitive lipase (HSL), which is well expressed in myeloid cells [4, 25]. This leads to a decrease in glycolysis, which is further supported by a decrease in mTOR kinase activity which is required for translation of glycolytic enzymes [18, 26]. Such an effect results in the upregulation of the Krebs’ cycle and thus in a decrease in intracellular 2-OG levels that affect HIF-1α PHD activity, thus preventing physiological degradation of HIF-1α protein. The effects described above are summarised in the scheme presented in the Figure 11.

Caffeine was revealed to inhibit mTOR, thus downregulating glycolysis. Previous reports suggested a possible influence of caffeine on NF-kB activity and calcium-dependent signalling [7, 27]. All these effects are likely to be responsible for the decreases in cytokine production observed in the present study (e.g. TNF-α and IL-6 in the case of TLR ligand stimulation and VEGF when the cells were exposed to SCF). These effects took place in both THP-1 and primary AML cells.

Despite the well-known fact that caffeine is an effective inhibitor of cAMP-PDE [2], no significant changes in intracellular cAMP levels were observed in THP-1 or AML cells. This could be explained by the fact that neither TLRs nor the SCF receptor (Kit) are G-protein coupled receptors. Therefore, they do not activate adenylate cyclase, the enzyme, which converts ATP into cAMP.

A different picture was observed in primary human basophils which did not express detectable amounts of XOD. Here, we also observed inhibition of IgE-induced mTOR kinase activity and abrogation of the mediator (histamine) release. However, HIF-1α accumulation did not seem to be PHD dependent in these cells and therefore, upon inhibition of mTOR kinase activity, IgE-induced HIF-1α accumulation was attenuated by caffeine. However, in contrast to AML and THP-1 cells, we observed a significant increase in intracellular cAMP levels in basophils which were further upregulated by caffeine. Basophils release histamine and also express H2-type histamine receptors which are G-protein-coupled and thus activate adenylate cyclase [15]. The cAMP levels induced by the autocrine actions of histamine are therefore preserved by caffeine as an inhibitor of cAMP-PDE. In this case, it is likely that we observed additional inhibition of mTOR activation due to the indirect actions of caffeine by increasing cAMP. All the effects reported were studied at 1 mM caffeine. However, dose-response investigations demonstrated that it displays its activities in primary AML cells and basophils at 0.1 mM though not at 0.01 mM.

Taken together, our results demonstrate, for the first time, that unmodified caffeine inhibits mTOR function by targeting its kinase activity during the biological responses of various human myeloid cells. These findings support observations made by other groups regarding caffeine effects on the mTOR pathway in non-blood cells [7, 8]. Furthermore, caffeine inhibits XOD activity but, unlike XOD-specific inhibitors, it does not upregulate the re-use of purines in AML cells. In contrast, basophils do not express detectable amounts of XOD. Since caffeine is a non-toxic drug with relatively few side effects at therapeutic concentrations, and easily metabolised in non-blood cells, our findings may lead to new avenues for therapy of human disorders affecting myeloid hematopoietic cells. However, it is obvious that high concentrations of caffeine would need to be maintained in order to achieve either its anti-inflammatory/anti-allergic effects or to affect SCF-induced biological responses. Because of this, the therapeutic actions of caffeine would more likely be achieved following external applications of this drug. For example, caffeine could be applied as a cream supplement or through other forms of direct application of unmodified caffeine on target myeloid cells with the purpose of their inactivation or elimination (previous studies have shown the ability of caffeine to induce programmed cell death [7, 8]). Alternatively, for internal applications, one might consider the recently discovered advantages of Nanobiotechnology or other highly specific forms of drug delivery in order to supply caffeine directly to myeloid cells.

## MATERIALS AND METHODS

### Materials

RPMI-1640 medium, foetal calf serum and supplements, caffeine, bovine liver XOD, PGN, goat anti-human-IgE, caffeine metabolites and an ATP luminometric detection kit were purchased from Sigma (Suffolk, UK). AMP/cAMP detection kits were purchased from Promega (Southampton, UK). Maxisorp™ microtitre plates were obtained from Nunc (Roskilde, Denmark) as well as from Oxley Hughes Ltd (London, UK). Mouse monoclonal antibodies to HIF-1α, mTOR and β-actin as well as rabbit polyclonal antibodies against phospho-S2448 mTOR and phospho-T2446 mTOR were from Abcam (Cambridge, UK). Antibodies against phospho-T389 p70 S6 kinase 1 (p70 S6K1), total and phospho-S65 eukaryotic initiation factor 4E binding protein 1 (eIF4E-BP1) antibodies were obtained from Cell Signalling Technology (Danvers, MA USA). Goat anti-mouse and goat anti-rabbit fluorescence dye-labelled antibodies were obtained from Li-Cor (Lincoln, Nebraska USA). ELISA-based assay kits for the detection of IL-6, TNF-α and VEGF were purchased from R&D Systems (Abingdon, UK). All other chemicals were of the highest grade of purity that were commercially available (from either Sigma or Fisher Scientific).

### THP-1 human myeloid cells

THP-1 human leukaemia monocytic macrophages were obtained from the European collection of Cell Cultures (Salisbury, UK). Cells were cultured in RPMI 1640 media supplemented with 10% foetal calf serum, penicillin (50 IU/ml) and streptomycin sulfate (50 μg/ml). Cells used in the experiments were from the 5–35^th^ passage.

### Primary human AML cells

Primary human AML mononuclear cells (AML-PB001F, newly diagnosed/untreated) were purchased from AllCells (Alameda, CA, USA) and handled in accordance with manufacturer's instructions. Cells derived from two different patients were used in the experiments.

### Primary human leukocytes obtained from healthy donors

Primary human leukocytes were obtained from buffy coat blood (prepared from healthy donors) purchased from the National Health Blood and Transfusion Service (NHSBT, UK) following ethical approval (REC reference: 12/WM/0319). Mononuclear-rich leukocytes were obtained by Ficoll-density centrifugation according to the manufacturer's protocol. Cell numbers were determined using a haemocy-tometer and diluted accordingly with HEPES-buffered Tyrode's solution before treatment as indicated.

### Primary human basophils

Primary human basophils were obtained from buffy coats and isolated from the leukocyte-rich fraction obtained by Ficoll density centrifugation as described above. Basophils were then purified by a negative selection procedure to 90–100% purity (as judged by alcian blue staining) using a commercially available kit, as previously described [28]. Basophils were pre-incubated for 15 min at 37°C in HEPES-buffered Tyrode's solution and then treated as indicated.

### Stem cell factor

Human SCF protein was produced in *E.Coli* and purified in accordance with published protocols [29].

### Western blot analysis

Expressions of HIF-1α, total and phospho-T389 p70 S6K1, total and phospho-S65 eIF4E-BP1 as well as XOD protein levels were determined by Western blot analysis and compared to β-actin in order to determine equal protein loading, as previously described [10]. Li-Cor goat secondary antibodies, conjugated with fluorescent dyes, were used according to the manufacturer's protocol in order to visualise the proteins of interest using a Li-Cor Odyssey imaging system. Western blot data were subjected to quantitative analysis using Odyssey software and values were normalised against respective β-actin bands.

### Detection of phospho-S2448 and phospho-T2446 mTOR in cell lysates by ELISA

Phosphorylation of mTOR was monitored using ELISA assays as recently described [10, 21, 30]. Briefly, the ELISA plates were coated with mouse anti-mTOR antibodies and blocked with 2% BSA. Cell lysates were then added to the wells and kept at room temperature for at least 2 h (under constant agitation). After extensive washing with TBST buffer, anti-phospho-S2448 (or anti-phospho-T2446) mTOR antibody was added and plates were incubated for at least 2 h at room temperature with constant agitation. Plates were then washed with TBST buffer and incubated with 1:1000 HRP-labelled goat anti-rabbit IgG in TBST buffer. After extensive washing with TBST, bound secondary antibodies were then detected by the peroxidase reaction (ortho-phenylenediamine/H_2_O_2_).

### HIF-1α PHD assay

To detect HIF-1α PHD activity we employed a peptide-based assay [31]. HIF-1α-free cell lysates were used to avoid the impact of intracellular HIF-1α hydroxylation. Lysates of non-treated and treated THP-1 cells were incubated for one hour in 96-well ELISA plates which were coated with HIF-1α capture antibodies and blocked with BSA as described previously. Lysates were then subjected PHD assay following this incubation period.

### Detection of intracellular ATP, cAMP, AMP, ROS and 2-OG levels, xanthine oxidase activity as well as glycolysis intensity

ATP was detected using a commercial luminometric kit (Sigma) according to the manufacturer's protocol. AMP and cAMP levels were detected using luminometric assay kits in accordance with manufacturer's protocols. Intracellular ROS levels were also monitored by luminometric assay as previously described [32]. 2-OG levels were analysed using a glutamate dehydrogenase spectrophotometric assay [33]. Xanthine oxidase activity was measured as described before [21]. The intensity of glycolysis was determined based on the ability cell lysate enzymes, used as a multienzyme preparation, to convert glucose into lactate under anaerobic conditions, which was achieved by employing an anaerobic chamber [10].

### Detection of IL-6, TNF-α and VEGF release

Concentrations of these cytokines released into the cell culture media were analysed by ELISA (R&D Systems assay kit) according to the manufacturer's protocol.

### Measurement of histamine release

Histamine releases were assessed using spectro-fluorometic autoanalysis as previously described [34]. Histamine releases were calculated from the histamine contents released into the supernatants as a percentage of total histamine content present in lysed cell pellets.

### High performance liquid chromatography

Caffeine and its metabolites were analysed in urine according to a previously described protocol [35, 36] with minor modifications. Healthy subjects were asked to refrain from all caffeine containing beverages and chocolate for 1 day. At 8 a.m. on the test day subjects were asked to consume 2 cups of very strong espresso (approximately 200 mg of caffeine) and 6 hours later urine was collected for analysis.

10 ml of urine were mixed with 200 mg of ascorbic acid. The pH was adjusted to 3.5 with 30% acetic acid. 200 μl of urine were mixed with 140 mg of ammonium sulfate, 25 μl of internal standard (137 MU) and vortexed at full speed for 1 min. The metabolites were extracted with chloroform:isopropanol (95:5) and centrifuged. The organic phase was evaporated to dryness using nitrogen or Speedvac and the pellet was dissolved in 750 μl of diluent containing MeOH : acetic acid : water (20:0.5:979.5 vol:vol:vol) and 5 μl of the sample was analysed by Agilent HPLC system (the flow rate was 0.5 ml/min) with a UV detector using a gradient elution profile. Caffeine, caffeine metabolites and the internal standard were separated on an Agilent XBD C18 reverse phase column (1.8 μm, 4.6 × 50 mm). The solvents used for the elution were mobile phase A containing MeOH : acetic acid : water (20:0.5:979.5 vol:vol:vol) and mobile phase B (MeOH : acetic acid : water 700:0.5:229.5 vol:vol:vol). The gradient program was 0% B (0 min), 100% (0.1 to 15 min) and 0% (15.1 to 18 min). The analytes were detected by UV absorbance at 280 nm. Calibration curves were developed using known amounts of metabolites.

### Analysis of total cytochrome P450 and activity of CYP450 1A2

Total CYP450 was analysed by a classic spectrophotometric method initially proposed by Omura and Sato [37]. CYP450 1A2 activity was analysed based on its ability to oxidise caffeine using NADPH as a donor. Mouse liver homogenates obtained from commercial sources were used as positive control.

### Statistical analysis

Each experiment was performed at least three times and statistical analysis was conducted using a two-tailed Student's *t* test. Statistical probabilities (p) were expressed as *, where *p* < 0.01.

## SUPPLEMENTARY FIGURES


