# Supplementary material for: Caffeine affects the biological responses of human hematopoietic cells of myeloid lineage via downregulation of the mTOR pathway and xanthine oxidase activity
Source: Oncotarget. 2015 Sep 11;6(30):28678–92. doi: 10.18632/oncotarget.5212 (PMC4745685; doi:10.18632/oncotarget.5212)
Supplement: Supplementary file 1 [file oncotarget-06-28678-s001.pdf]

SUPPLEMENTARY FIGURES

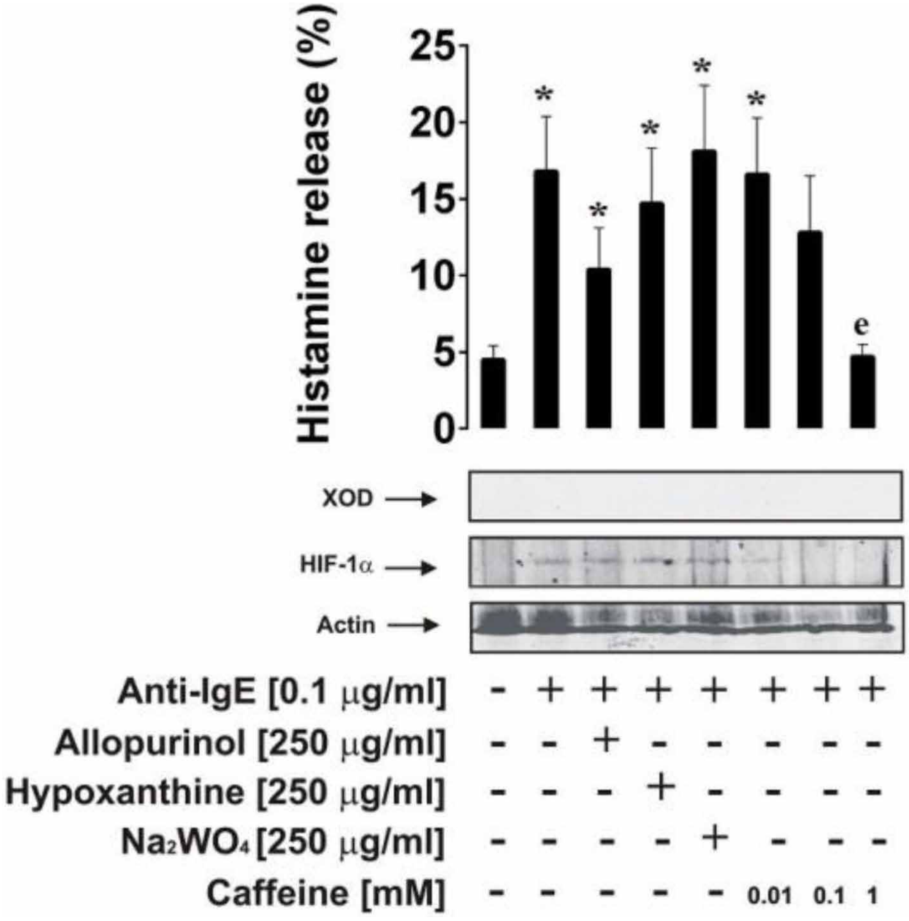

Supplementary Figure S1: Effects of allopurinol, hypoxanthine, sodium tungstate and caffeine on anti-IgE-induced histamine release and HIF-1α accumulation in primary human basophils. Basophils were exposed for 2 h to 0.1 μg/ml anti-IgE with or without 30 min pre-treatment with indicated concentrations of inhibitors or hypoxanthine. Western blot data show one representative experiment of three that gave similar results. Quantitative data are shown as means ± S.D ; * – $p < 0.01$ vs. control ($n = 3$), e – $p < 0.01$ vs. anti-IgE alone.

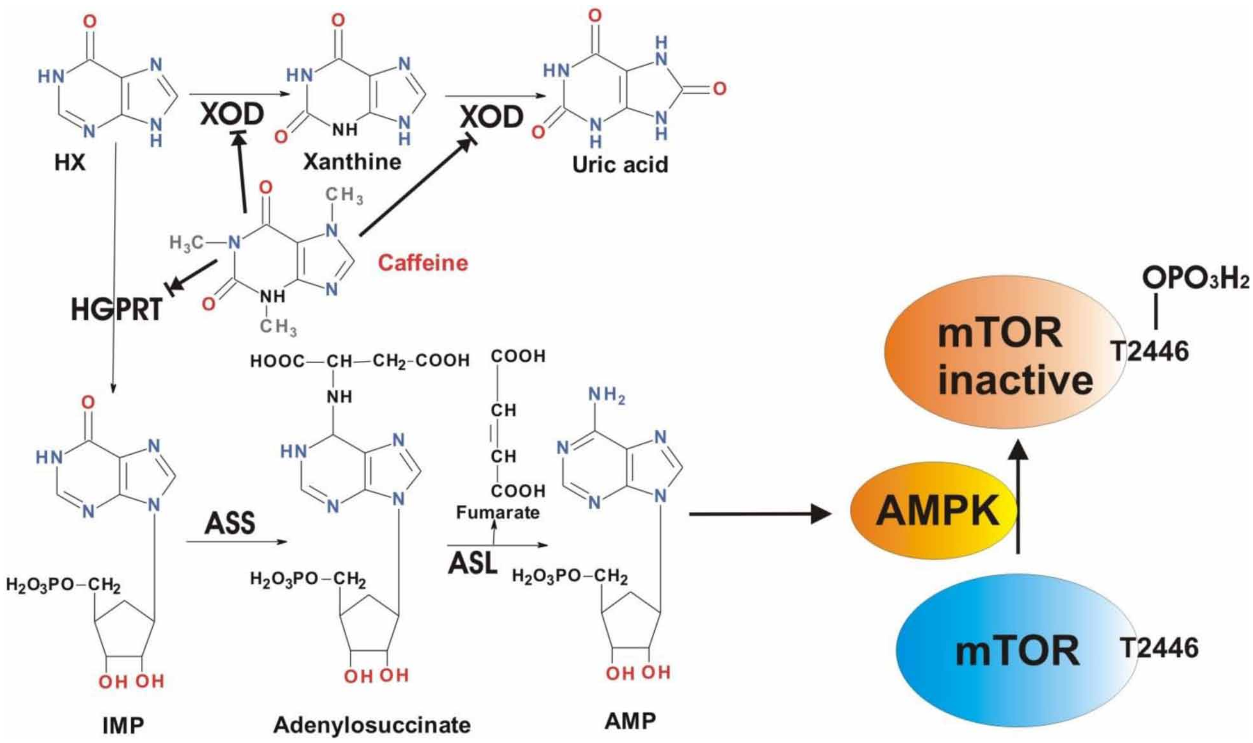

Supplementary Figure S2: Possible biochemical mechanisms of the actions of caffeine on hypoxanthine degradation and re-use. Additional abbreviations used: ASS – adenylosuccinate synthase; ASL – adenylosuccinate lyase.
